# The changing epidemiology worldwide of *Mycobacterium ulcerans*

**DOI:** 10.1017/S0950268818002662

**Published:** 2018-10-08

**Authors:** D. P. O'Brien, I. Jeanne, K. Blasdell, M. Avumegah, E. Athan

**Affiliations:** 1 Geelong Centre for Emerging Infectious Diseases, Geelong, Australia; 2 Department of Medicine and Infectious Diseases, Royal Melbourne Hospital, University of Melbourne, Melbourne, Australia; 3 Medecins Sans Frontieres, London, UK; 4 School of Medicine, Deakin University, Geelong, Australia; 5 CSIRO Health and Biosecurity, Australian Animal Health Laboratory, Geelong, Australia

**Keywords:** Buruli ulcer, epidemiology, *Mycobacterium ulcerans*, spread, transmission

## Abstract

*Mycobacterium ulcerans* is recognised as the third most common mycobacterial infection worldwide. It causes necrotising infections of skin and soft tissue and is classified as a neglected tropical disease by the World Health Organization (WHO). However, despite extensive research, the environmental reservoir of the organism and mode of transmission of the infection to humans remain unknown. This limits the ability to design and implement public health interventions to effectively and consistently prevent the spread and reduce the incidence of this disease. In recent years, the epidemiology of the disease has changed. In most endemic regions of the world, the number of cases reported to the WHO are reducing, with a 64% reduction in cases reported worldwide in the last 9 years. Conversely, in a smaller number of countries including Australia and Nigeria, reported cases are increasing at a rapid rate, new endemic areas continue to appear, and in Australia cases are becoming more severe. The reasons for this changing epidemiology are unknown. We review the epidemiology of *M. ulcerans* disease worldwide, and document recent changes. We also outline and discuss the current state of knowledge on the ecology of *M. ulcerans*, possible transmission mechanisms to humans and what may be enabling the spread of *M. ulcerans* into new endemic areas.

## Background

**Fig. 1.**
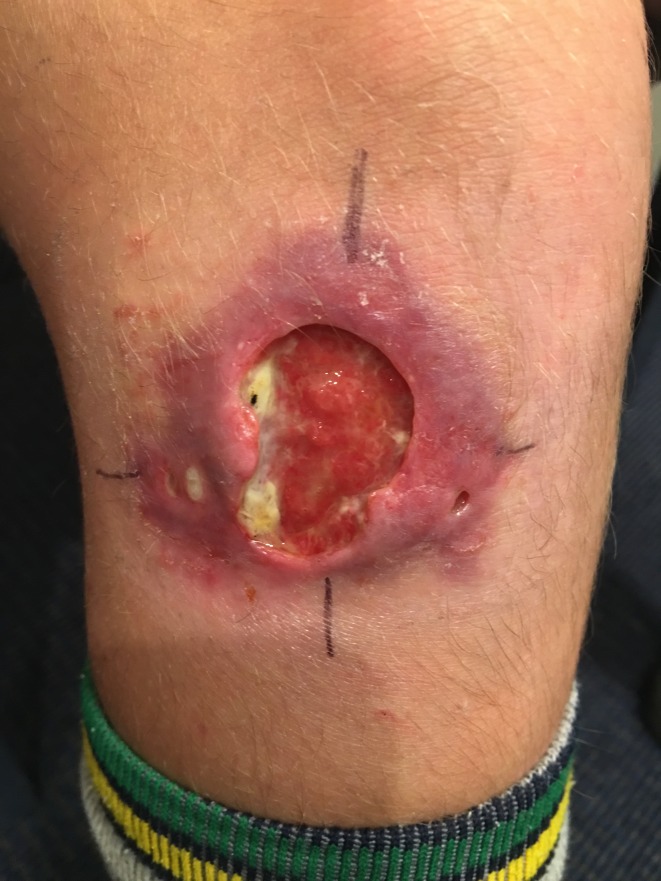
A severe *Mycobacterium ulcerans* lesion on the knee of an 11-year-old boy.

*Mycobacterium ulcerans* is a slow-growing organism that causes necrotising infections of skin and soft tissue and is classified as a neglected tropical disease by the World Health Organization (WHO) [1]. Ulcers are the most common form of disease, but it can also manifest as a subcutaneous nodule, plaque or as a diffuse and aggressive oedematous form, and can be complicated by osteomyelitis [2–4] (Fig. 1). The disease is known internationally as Buruli ulcer (BU) after the county in Uganda where cases were described in the 1960s. Previously, wide surgical excision was the treatment of choice [5], but dual antibiotic combinations have recently been shown to be highly effective at curing lesions [6, 7] and are now the recommended first-line treatment [2, 8]. Surgery is used to aid wound healing and prevent deformity, or if antibiotics are not tolerated, contraindicated or declined [8]. If diagnosed and treated early, outcomes are excellent, but if left untreated, the disease can progress resulting in high levels of morbidity and permanent disability [4, 9].

In Africa, the disease affects mainly children [4] with more than 50% of cases occurring in those 5 to <15 years of age. Conversely in Australia, it affects mainly adults, with a median age of about 60 years [10]. Nevertheless it can occur in all age groups, is found in males and females equally [4, 11, 12], and in Africa it commonly affects those living in remote areas with limited access to health care [4, 13]. The majority of people affected are overtly immunocompetent, though there appears to be an increased risk in those who are HIV-positive [14], and those who are immune suppressed are at risk of developing more severe disease [3, 15]. In Africa, the disease occurs most commonly in rural and resource-limited settings where access to safe water and sanitation is low, whilst in Australia, it occurs in high-income settings with access to high-level sanitation and treated water. Sero-epidemiological surveys in Africa suggest that only a small proportion of those exposed to *M. ulcerans* develop disease [16].

## Emergence of BU worldwide

**Fig. 2.**
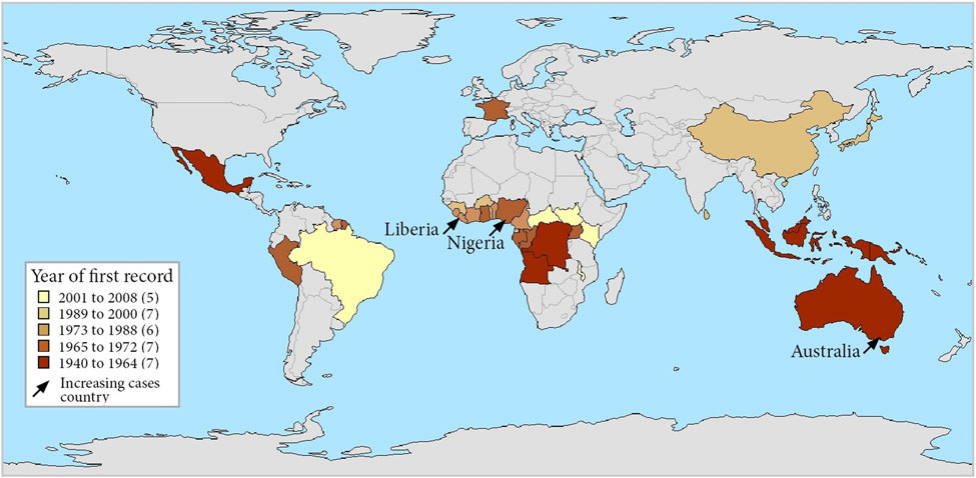
Map of countries reporting Buruli ulcer cases, stratified by year of first report. Note that each country is represented by its administrative area and that Buruli ulcer did not occur throughout each country. France is represented for its overseas department French Guiana – there has been no case in metropolitan France.

Although an outbreak of skin ulcers resembling BU was reported from Uganda in 1897, the first confirmed reports of BU were from Australia in 1948 in patients residing in the Bairnsdale region in south-eastern Victoria, where it is known as the Bairnsdale ulcer [17]. It has now been reported from 33 countries [1] and is the third most common mycobacterial disease worldwide in immunocompetent people after tuberculosis and leprosy [18] (Table 1 and Fig. 2). Confirmed cases of Buruli were then reported from three countries in the 1950s; Democratic Republic of Congo (DRC) [19], Mexico [20] and Uganda [21]. Nine countries across three continents reported their first cases in the 1960s; in Central Africa (Angola [20], Congo [22] and Gabon [23]), in West Africa (Nigeria) [20], in Asia and the Pacific (Papua New Guinea [24], Malaysia [25] and Indonesia [20]) and in South America (Peru [20] and French Guiana [20]). During the 1970s, cases were reported from West Africa for the first time (Benin [20], Ghana [26], Sierra Leone [20] and Cameroon [27]). In the 1980s, Ivory Coast [28] and Liberia [29] were added to the list of West African countries recording their first cases, along with Japan [30], Kiribati [31] and Suriname [32]. In the 1990s, four countries were added from West Africa (Burkina Faso [33], Equatorial Guinea [20], Togo [34] and Guinea [20]) as well as Sri Lanka [20] and China [35] from Asia. In the 2000s, Brazil reported cases for the first time [36] in addition to the East and Central African countries Kenya [37], Malawi [20], South Sudan [20] and Central African Republic [18].

**Table 1.** Countries with published reports of Buruli ulcer cases including year of initial report and changes in numbers of cases reported over time

Country	Year cases first reported and reference	Year of peak disease cases reported to WHO 2002–2016	Peak number of cases reported to WHO in a year	2016 cases reported to WHO	Percentage change in 2016 from peak reported cases in a year
Angola	1960 [20]	NA	NA	NA	–
Australia	1940 [17]	2016	186	186	Peak
Benin	1977 [20]	2007	1203	312	−74%
Brazil	2007 [36]	NA	NA	NA	–
Burkina Faso	1998 [33]	NA	NA	NA	–
Cameroon	1973 [27]	2004	914	85	−91%
Central African Republic	2008 [18]	2008	3	NA	–
China	1997 [35]	NA	NA	NA	–
Congo	1966 [22]	2006	370	NA	–
Democratic Republic of Congo	1950 [19]	2004	487	175	−64%
Equatorial Guinea	1998 [20]	2005	3	NA	–
French Guiana	1969 [20]	2002	27 [38]	NA	–
Gabon	1968 [23]	2005	91	39	−57%
Ghana	1971 [26]	2006	1048	371	−65%
Guinea	1993 [20]	2006	279	72	–74
Indonesia (not confirmed)^a^	1960 [20]	NA	NA	NA	–
Ivory Coast	1980 [28]	2009	2679	376	−86%
Japan	1989 [30]	2011,2013	10	2	−80%
Kenya	2008 [37]	NA	NA	NA	–
Kiribati (not confirmed)	1987 [31]	NA	NA	NA	–
Liberia	1981 [29]	2015	105	NA	–
Malawi (not confirmed)^a^	2001 [20]	NA	NA	NA	–
Malaysia	1964 [25]	NA	NA	NA	–
Mexico	1953 [20]	NA	NA	NA	–
Nigeria	1967 [20]	2016	235	235	Peak
Papua New Guinea	1962 [24]	2004	31	16	–48
Peru	1969 [20]	NA	NA	NA	–
Sierra Leone	1975 [20]	2011	28	NA	–
South Sudan	2001 [20]	2002	568	NA	–
Sri Lanka (not confirmed)^a^	1992 [20]	NA	NA	NA	–
Suriname	1984 [32]	NA	NA	NA	
Togo	1996 [34]	2004	800	83	−90%
Uganda	1958 [21]	2002	117	NA	

a The presence of *M. ulcerans* was not microbiologically confirmed in this report.

## Current epidemiological situation

The greatest burden of disease is found in West and Central Africa where the highest number of cases are reported from Cote D'Ivoire [39], Benin [40], Ghana [41], Cameroon [42] and the DRC [43]. Estimated incidence rates include 21.5 per 100 000/year in parts of Benin [40] and 20.7 per 100 000/year in Ghana overall with up to 158.8 per 100 000/year in some affected districts [41]. Cases continue to be reported from South America (mainly in French Guiana) [38, 44, 45], Asia and the Pacific (mainly in Papua New Guinea) [18,20]. BU is predominantly found in tropical and subtropical climates, apart from south-eastern Australia [12, 46] (with estimated incidence rates of up to 404 per 100 000/year) [47], China and Japan [48] (Table 1). In Australia, it has also been reported from tropical areas in Queensland, where it is known as the Daintree ulcer [49–51], and in the Northern Territory [52]. Importantly, case numbers reported by countries may be influenced by political stability, access to health care, funding for case detection activities, quality of reporting systems and availability of diagnostics. For example, an exhaustive field survey conducted in DRC involving more than 39 000 households showed that only 7% of active BU cases were captured in the hospital-based reporting system [53].

**Fig. 3.**
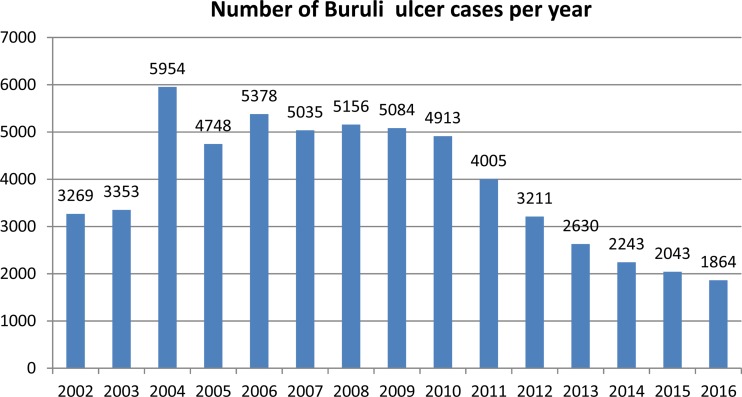
Number of Buruli ulcer cases worldwide reported to the WHO from 2002 to 2016.

In recent years, the number of disease cases reported to the WHO worldwide has been steadily decreasing; from 5156 cases in 2008 to 1864 cases in 2016 – a reduction of 64% (Fig. 3). This mainly reflects reductions in Africa where there has been a decline in most of the highest prevalence countries (Table 1). For example, Cote D'Ivoire reported 2679 cases in 2009 and only 376 cases in 2016 (86% reduction), Ghana's reported cases reduced from 1048 in 2010 to 371 in 2016 (65% reduction) and Benin's from 1203 cases in 2007 to 312 in 2016 (74% reduction). An African country going against the trend is Nigeria, where cases were first reported in 2009 and have increased from 24 in that year to 235 in 2016 (879% increase). Case incidence reported from French Guyana has also decreased from 6.07 cases per 100 000 person-years in 1969–1983 to 4.77 cases per 100 000 person-years in 1984–1998 and to 3.49 cases per 100 000 person-years in 1999–2013 [38].

Conversely, in Australia, the number of reported cases has been increasing with 186 reported in 2016 compared with 42 in 2010 (343% increase). This mainly reflects a rapidly increasing number of cases reported from the coastal regions of the south-eastern state of Victoria where there has been a 248% increase in cases in the last 4 years (79 cases in 2014 to 275 in 2017). In this region, the disease has emerged in new geographical areas including the Mornington Peninsula outside of Melbourne, and the proportion of cases presenting with severe disease has doubled since 2010 [10]. Paradoxically, in two adjacent peninsulas separated by only a few kilometres of ocean with similar climate and resident populations, there are diverging epidemics – increasing case numbers on the Mornington Peninsula and reducing case numbers on the Bellarine Peninsula [54].

## Ecology of BU

Evidence indicates that *M. ulcerans* likely evolved from *M. marinum* by acquiring a virulence plasmid that produces its pathogenic mycolactone toxin [55] and allowed it to adapt to a specific environmental niche [56]. Laboratory conditions that favour the growth of *M. ulcerans* are low oxygen [57], relatively low temperatures (28–33 °C) [58, 59], moderately acidic environments (pH 5.4–7.4) [60] and low levels of ultra violet rays [58]. This may explain why *M. ulcerans* is often found at the bottom of aquatic habitats or protected by biofilms [61]. However, despite extensive research, the environmental reservoir of the organism and mode of transmission of the infection remain unknown. A major factor limiting this understanding is that the organism can rarely be cultured from the environment [62], although PCR testing of water, aquatic plants, soil and detritus from swamps can show evidence of *M. ulcerans* [47, 63–67].

In endemic areas, the disease is highly focal with endemic and non-endemic areas separated by only a few kilometres [12, 13]. It is usually associated with wetlands, especially those with slow-flowing or stagnant waters such as floodplains or swampy areas [13, 65]. Studies have suggested that farming activities close to rivers [39] and swimming in rivers in endemic areas [68] are risk factors for acquisition of BU. The construction of dams and irrigation systems have also been associated with increased cases [69], although in French Guyana, a reduction in cases has been observed, likely related to reduced flooding of downstream districts [70]. A major process of land-use change, deforestation, is known to result in increased erosion, which has been speculated to result in run-off contamination of water bodies with *M. ulcerans* [71]. Deforestation has also been found to alter the composition of freshwater communities in French Guiana, impacting the abundance of *M. ulcerans* [72].

In Victoria, Australia, native and domestic mammals including possums, dogs, cats, koalas, horses and alpacas have developed disease [47], but whether they are intimately involved in transmission, or accidental hosts, remains unclear. Outside of Australia, *M. ulcerans* has rarely been detected in vertebrates, although lesions on a wild mouse (*Mastomys* sp.) from Ghana [73] and on a goat and dog from Benin have been found PCR-positive for *M. ulcerans* [74], as have domestic duck faeces in Cameroon and wild agouti faeces in Ivory Coast [75, 76]. In contrast, a range of aquatic invertebrates from numerous taxa representing several orders have been found positive for *M. ulcerans* DNA from many locations in Africa [65, 77].

Recent evidence from Australia suggests that whatever the source in the environment, it may only persist for a short time [78]. In 21 patients with *M. ulcerans* who were part of a family cluster, the median time to diagnosis between family members was 2.8 months, and none were diagnosed more than 23 months apart in a cohort spanning 18 years and nearly 2000 combined years of elapsed time since diagnosis. This suggests that in this setting the exposure risk is short term, and thereafter diminishes. Environmental studies in Cameroon found that samples from a water hole used by local people remained PCR-positive for more than 2 years, and at least 12 months after all local human *M. ulcerans* disease cases were treated and cured [75]. This suggests that although the risk of disease transmission has diminished, the organism may continue to persist for longer periods in some environments.

## Spread of BU into new areas

The mechanism by which *M. ulcerans* is introduced into new areas is unknown. However in Australia, research using population genomics suggests that the organism has moved from east to west in the southern state of Victoria, and that this relates to the introduction and expansion of *M. ulcerans* into new environments rather than an awakening of quiescent pathogens [79]. Also by analysing the population genomics of isolates from 11 different countries in Africa, Vandelannoote *et al*. concluded that the spread of *M. ulcerans* across Africa was a relatively modern phenomenon and one that had escalated since the late 19th and the early 20th centuries [80]. Their work suggested human-induced changes and activities were behind the expansion of *M. ulcerans* in Africa with humans with active BU lesions inadvertently contaminating aquatic environments during water contact activities.

In Australia, it is possible that the dispersal of possums or their active transfer by humans from one area to another may promote the introduction of *M. ulcerans* into new areas. Urban development may also increase the disease risk because possums can reach high population densities in remaining refuge habitats (e.g. parks, ‘bush-style’ gardens) due to their generalist nature and ability to utilise human environments and food sources [81, 82]. Regardless of whether they are directly involved in introducing disease, as possums themselves develop BU, they could act as sentinel animals for detecting the emergence of *M. ulcerans* disease in new areas in Australia [83].

## Transmission of BU to humans

Insects such as mosquitoes [84, 85] and aquatic biting arthropods [59, 86] have been proposed as vectors for transmission, but this remains an open question [66]. Mosquitoes in Australia have tested positive for *M. ulcerans* by PCR [85] and there are epidemiological links such as the use of insect repellent on exposed body surfaces and the use of mosquito nets being associated with a reduction in *M. ulcerans* incidence [84, 87]. Additionally, in Australian towns in an endemic area, a strong dose–response relationship was found between the detection of *M. ulcerans* in mosquitoes and the risk of human disease [88]. Possible mechanisms for infection may involve direct inoculation of the organism under the skin via a bite, as suggested by a recent study showing that if the skin already surface contaminated with *M. ulcerans* is subjected to a puncturing injury in the form of a needle or a bite from a live mosquito, then *M. ulcerans* lesions can develop at the puncture site [89]. Alternatively, infection may result from a bite leading to a wound which is secondarily infected by *M. ulcerans* from environmental sources such as soil. Although arguing against this is evidence from a guinea pig model where applying *M. ulcerans* organisms directly to abraided skin did not establish infection – instead infection was only established when organisms were inoculated under the skin [90]. It has been proposed that mosquitoes carry the organism on their proboscis following contact or feeding with contaminated environmental sources and then directly transmit it through their bite [85], although the widespread nature and potential travel of mosquitoes both inside and outside the restricted geographic regions affected by BU argues against this. Nevertheless, it is possible that mosquito movements between the affected and unaffected areas may be limited as some implicated mosquito species, such as *Aedes notocsriptus*, have short flight distances and low dispersal ability [91], whilst specific larval habitat requirements can also restrict distribution [92]. If affected mosquito populations in BU endemic areas were relatively isolated from other populations, this could result in the geographic restriction observed.

The strongest evidence for a zoonosis comes from Australia involving native mammal species; the common ringtail (*Pseudocheirus peregrinus*) and common brushtail (*Trichosurus vulpecula*) possums. Research has found that 19% of these animals in an endemic area (Point Lonsdale on the Bellarine Peninsula) had *M. ulcerans* clinical disease, whilst a further 14% were asymptomatic but had high levels of *M. ulcerans* DNA detected on PCR examination of their faeces [47]. In addition, the location, proportion and concentration of *M. ulcerans* DNA in possum faeces strongly correlated with that of human *M. ulcerans* disease cases in at least two outbreaks where it has been measured: Sorrento on the Mornington Peninsula [83] and Point Lonsdale on the Bellarine Peninsula [47]. Additionally, in nearby areas with no cases of human disease possum faeces were not found to contain *M. ulcerans* DNA [47]. It is theoretically possible that infected possums amplify the organism in the environment [93], leading to an increased risk of infection via contact with a contaminated environment, or an intermediate vector such as a mosquito could mechanically transmit the bacteria from infected possums to humans via a bite. Further research is required to investigate these potential transmission mechanisms and determine if possums play a pivotal role in the transmission of disease to humans or whether they are simply accidental hosts.

In Africa, *M. ulcerans* has been detected by PCR in aquatic insect species in the order Hemiptera (families: Naucoridae and Belostomatidae), which are known to bite humans, suggesting that transmission may occur through these bites [59]. This is supported by the detection of *M. ulcerans* in the salivary glands of these insects after eating snails containing the organism [94], and the finding that it can also be transmitted to laboratory mice via their bite [86]. In an outbreak in Philip Island, Australia, it was postulated that aerosols generated by spray irrigation using contaminated water may have disseminated *M. ulcerans* and infected humans via the respiratory tract, or through contamination of skin lesions and minor abrasions [95], but this has not been proven.

Another recent study on an Australian cohort [78] confirms previous suggestions that human-to-human transmission does not occur [96]. Although cases were often clustered amongst families (6.5% of cases had another family member affected), the short time period between the diagnosis of family clustered cases (median 2.8 months) was shorter than the estimated incubation period of the disease (4.5 months) [78]. Additionally, whole genome SNP analysis of isolates from three paired family clusters revealed isolates derived from two of the three family clusters were not genetically identical and family cluster isolates were not any more genetically related than those of six random isolates from the same geographic region [78].

The location of clinical *M. ulcerans* lesions provides some information about possible transmission mechanisms. A study of 649 lesions in 579 Australian patients revealed that most lesions were on exposed body areas, notably upper and lower limbs, and were commonly over a joint. Few lesions were found on the head and neck, palms of hands, soles of feet and trunk [97]. Furthermore, the distribution was non-random, with a strong predilection for ankles, elbows and calves. Differences in the pattern of lesion distribution were also found between genders (men had more lesions on upper limbs and less on lower limbs than women), age groups (those aged ⩾65 years were less likely to have proximal upper limb lesions compared with those <65 years) and season of likely acquisition (lesions on the arm and shoulder were more common amongst those likely acquired in the warmer months). Age, gender and seasonal differences may relate to exposure risk via such mechanisms as trauma, insect bites or soil contact relating to differences in clothing worn or activities undertaken. Similar findings have also been reported from a smaller study in Cameroon [98]. Case–control studies in Africa have also identified wearing short lower body clothing whilst farming as risk factors for *M. ulcerans* [99] and covering limbs during farming as protective for *M. ulcerans* [100]. These findings suggest that *M. ulcerans* transmission and pathogenesis may be similar across the world despite very different geographical and climatic conditions.

Transmission also appears to be seasonal. In Australia, the large majority of cases (>70%) are likely acquired in the warmest 6 months of the year [97], and in Cameroon, the likely time of infection was seasonal and highest between the months of August and October [101]. Studies have also reported an association with rainfall; studies from Ghana and Cameroon report that the proportion of *M. ulcerans*-positive samples from the environment was higher during the months with higher rainfall levels than during the dry season months [101, 102] and reports from French Guyana [70] and Australia suggest an increase in cases associated with periods of high rainfall followed by dry periods [103].

## Future prospects

The epidemiology of *M. ulcerans* has clearly changed over time and is expected to continue evolving into the future. Although the explanations for this are not fully understood, the processes associated with increasing anthropogenic land-use change such as deforestation, road construction, flooding and population settlement may have significant impacts on this environmental pathogen, affecting both its future distribution and human exposure risk [93]. For example, the increase in cases in recent years observed in south-eastern Victoria, Australia, may be reflective of the impact of new residential developments which may have altered the environment and impacted both aquatic and terrestrial communities, including a proposed reservoir of *M. ulcerans*, the possum.

Flooding, through environmental disturbance and contamination of aquatic habitats, has regularly been linked to outbreaks of BU [93]. As dams alter the degree and frequency of downstream flooding, the increase in dam building occurring in many regions where BU is endemic, may also alter the distribution of this pathogen. Climate change will likely also be influential, through altered temperatures, increased frequency of extreme weather events and intense flooding events, and inundation of coastal foci, such as the Victorian hot-spot, through changes in sea level. The increased mobility of today's societies may additionally help to modify the distribution of *M. ulcerans*, by altering the genetic variants of this pathogen present in an area (as found in the Offin river valley in Ghana) [104], and by providing opportunities for the establishment of new foci where suitable environmental conditions exist.

## Conclusions

The reasons for the changing epidemiology of BU worldwide are unknown. Possibilities include changing environmental conditions such as rainfall and temperature in the era of climate change, changes in population dynamics and land use, improved sanitation and access to healthcare or reduction in exposure through such things as increasing mosquito net use, or spill-over into humans from epidemics in animal reservoirs. Additionally, if humans represent the disease reservoir, it has been speculated that a reduced burden of disease in humans through improved case detection and increasing antibiotic use may be responsible for the reduction in cases in Africa [80]. However, the situation of increasing cases in Australia, where there is good access to medical care and antibiotic treatment is widely used argues against this explanation. An alternative explanation may be that improvements in the accuracy of diagnosis since PCR confirmation was introduced have reduced over-reporting of cases [105].

Importantly, cases of disease have been decreasing in most countries in recent years indicating a hopeful, positive trend. However, despite recent insights, it still has not been conclusively determined how this pathogen circulates in the environment, or how transmission to humans occurs. This requires the availability of robust scientific knowledge acquired by a thorough and exhaustive examination of the environment, local fauna, human behaviour and characteristics, and the interactions between them [106]. Only with this knowledge can control strategies or early warning systems be designed and implemented that effectively and consistently prevent the spread and reduce the incidence of this disease.
